# Cell Cycle Progression Regulates Biogenesis and Cellular Localization of Lipid Droplets

**DOI:** 10.1128/MCB.00374-18

**Published:** 2019-04-16

**Authors:** André L. S. Cruz, Nina Carrossini, Leonardo K. Teixeira, Luis F. Ribeiro-Pinto, Patricia T. Bozza, João P. B. Viola

**Affiliations:** a Program of Immunology and Tumor Biology, Brazilian National Cancer Institute (INCA), Rio de Janeiro, RJ, Brazil; b Laboratory of Immunopharmacology, Oswaldo Cruz Institute, FIOCRUZ, Rio de Janeiro, RJ, Brazil; c Program of Molecular Carcinogenesis, Brazilian National Cancer Institute (INCA), Rio de Janeiro, RJ, Brazil

**Keywords:** cell cycle, cellular transformation, lipid droplets, PLIN2

## Abstract

Intracellular lipid accumulation has been associated with a poor prognosis in cancer. We have previously reported the involvement of lipid droplets in cell proliferation in colon cancer cells, suggesting a role for these organelles in cancer development. In this study, we evaluate the role of lipid droplets in cell cycle regulation and cellular transformation. Cell cycle synchronization of NIH 3T3 cells revealed increased numbers and dispersed distribution of lipid droplets specifically during S phase. Also, the transformed cell lineage NIH 3T3-H-*rasV12* showed an accumulation of both lipid droplets and PLIN2 protein above the levels in NIH 3T3 cells. *PLIN2* gene overexpression, however, was not able to induce NIH 3T3 cell transformation, disproving the hypothesis that *PLIN2* is an oncogene. Furthermore, positive PLIN2 staining was strongly associated with highly proliferative Ki-67-positive areas in human colon adenocarcinoma tissue samples. Taken together, these results indicate that cell cycle progression is associated with tight regulation of lipid droplets, a process that is altered in transformed cells, suggesting the existence of a mechanism that connects cell cycle progression and cell proliferation with lipid accumulation.

## Abstract (precis)

Intracellular lipid accumulation has been associated with a poor prognosis in cancer. We have previously reported the involvement of lipid droplets in cell proliferation in colon cancer cells, suggesting a role for these organelles in cancer development.

## INTRODUCTION

Lipid droplets are cytoplasmic lipid-rich organelles involved in lipid storage. Though associated mainly with lipid accumulation and transport in the past, lipid droplets are now considered dynamic and ubiquitous organelles with roles in cellular signaling and the production of inflammatory mediators (1–3). These biological processes are thought to be linked not only to the lipid composition of lipid droplets but also to their diverse assortment of proteins, comprising, among others, enzymes involved in lipid metabolism and eicosanoid production (4, 5), Rab GTPases (6–8), and protein kinases (9–12). Lipid droplets also contain one group of proteins that plays a fundamental role in their formation, maintenance, modification, and involution: the PAT family of lipid droplets, named after its best-known members, *p*erilipin, *a*dipose differentiation-related protein (ADRP; also known as perilipin-2 [PLIN2] or adipophilin), and *T*IP47 (13, 14). In turn, PLIN2 is one of the most studied proteins within the PAT family, and while its biological function has not been completely elucidated, several lines of evidence point to a role in fatty acid uptake and neutral lipid storage (15–17), whereas PLIN2 overexpression is associated with increased lipid droplet biogenesis and triacylglycerol content (18, 19). Also, PLIN2 is ubiquitously expressed and is considered a marker of lipid droplet accumulation in the tissues of many species (20).

As the biology of lipid droplets is being uncovered, so is their relevance in several human pathologies. Increased lipid droplet biogenesis has been described in economically relevant diseases, such as diabetes (21, 22), atherosclerosis (23–25), and inflammatory processes (26–31), suggesting these organelles as potential targets for new therapies against these widespread illnesses. Likewise, increased numbers of lipid droplets have been observed in neoplastic processes, including colon adenocarcinoma (32), hepatocellular carcinoma (33), clear cell renal carcinoma (34), glioblastoma (35), and Burkitt lymphoma (36). Moreover, upregulated lipogenesis has been associated with a poor prognosis in breast, prostate, and colon cancers (37, 38). This evidence supports the possibility that lipid droplets are involved in the events of cellular transformation.

Following this line of thought, our group investigated the occurrence and function of lipid droplets in colon cancer, revealing a remarkable increase in lipid droplet numbers in human colon adenocarcinoma cell lines and human colon cancer biopsy specimens (32). Lipid droplets in colon cancer cells were described as active sites for enhanced prostaglandin E_2_ (PGE_2_) formation, whereas inhibition of lipid droplet biosynthesis abrogated PGE_2_ production and was correlated with decreased cell proliferation *in vitro*, implying that the increase in lipid droplet numbers could be associated with the high proliferation rate observed in these cancerous cells (32). Accordingly, recent work demonstrated that leptin-triggered activation of the mechanistic target of rapamycin (mTOR) in intestinal epithelial cells can drive intracellular lipid accumulation together with augmented cell cycle entry (39), revealing a mechanism that may contribute to obesity-enhanced susceptibility to colon carcinoma. These findings for colon cancer may implicate lipid droplets in cell cycle regulation. In parallel, other studies have supported the existence of a link between lipid droplets and cell cycle progression. Caveolin-1-deficient mice showed impaired liver regeneration after hepatectomy, caused by cell cycle arrest of hepatocytes due to their inability to accumulate lipid droplets efficiently (40). In budding yeast, hydrolysis of fat stored in lipid droplets by Tgl4 lipase is dependent on cell cycle regulatory machinery, and abrogation of Cdc28-mediated phosphorylation of Tgl4 causes a delay in cell cycle progression (41). Furthermore, simultaneous inhibition of lipid storage release and *de novo* fatty acid synthesis results in G_1_ cell cycle arrest in these organisms (41). Together, these studies suggest that continued cellular proliferation is dependent on the precise regulation of lipid droplets, and due to the importance of lipid droplets in cancer biology, the mechanisms that regulate their formation and their functional significance for tumorigenesis are now under intense investigation (2).

Uncontrolled proliferation is one of the hallmarks of cancer (42), and although several lines of evidence suggest that the increased lipid droplet biogenesis seen in cancer cells may contribute to cell proliferation, no definitive studies are presently available to establish a causal link between the increase in lipid droplet numbers and cell cycle progression. To address this question, we first analyzed the regulation of lipid droplets during the progression of nontransformed cell lines through the cell cycle. Then we evaluated whether oncogenic transformation is able to alter the regulation of lipid droplets in the cell cycle. By modulating the accumulation of PLIN2 protein in nontransformed cells, we analyzed the effects of lipid droplet biogenesis on cellular proliferation, as well as its transformation potential. Finally, we determined the expression pattern of PLIN2 protein in highly proliferative human colon cancer tissues.

## RESULTS

### Increased numbers and dispersed localization of lipid droplets are observed in synchronized cells during cell cycle progression.

**FIG 1:**
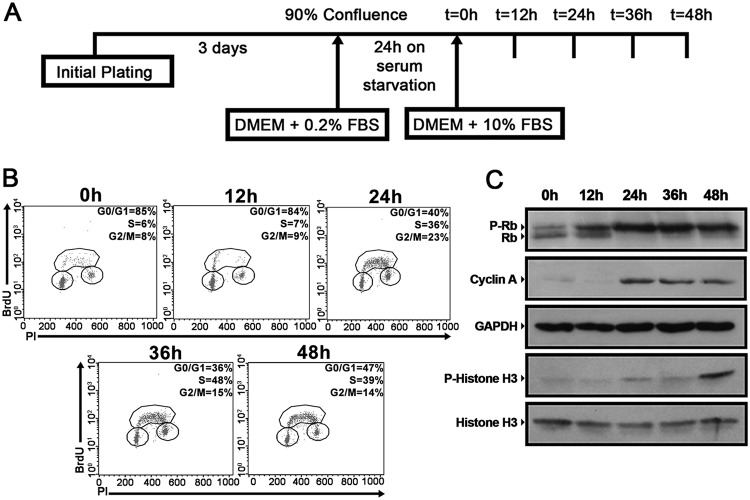
Synchronized NIH 3T3 cells progress uniformly through the cell cycle upon serum supplementation. (A) Experimental scheme of NIH 3T3 cell synchronization. NIH 3T3 cells were cultured for 72 h until 90% confluence and were then maintained on serum starvation for 24 h. The cells were subsequently replated at a low density, supplemented with a medium containing 10% FBS, and analyzed at the indicated time points. (B) Synchronized NIH 3T3 cells were incubated with 20 μM BrdU for 30 min before fixation at the indicated time points. Later, cell cycle analysis was performed using FITC-conjugated anti-BrdU antibodies together with propidium iodide staining. The percentage of cells in each stage of the cell cycle (G_0_/G_1_, S, and G_2_/M) is indicated. (C) Whole-cell extracts were prepared from synchronized NIH 3T3 cells at the indicated time points and were separated by SDS-PAGE. Western blotting was performed using antibodies to the indicated proteins. All data are representative of the results of at least three independent experiments.

Synchronization of proliferating cells is a widely used practice for studying the mechanisms that regulate cell cycle entry and progression (43). To evaluate the regulation of lipid droplets through the cell cycle, we synchronized NIH 3T3 murine fibroblasts by combining contact inhibition and serum starvation, as shown schematically in Fig. 1A. After this procedure, it was possible to observe an arrest of cell cycle progression, with an accumulation of cells at the G_0_/G_1_ phases and a low proportion of cell death (data not shown). Following synchronization, cells were replated at a low density and were supplemented with a medium containing 10% fetal bovine serum (FBS) to stimulate proliferation. It was possible to follow the reentry of synchronized NIH 3T3 cells into the cell cycle after serum supplementation by evaluating 5-bromo-2'-deoxyuridine (BrdU) incorporation along with propidium iodide (PI) staining (Fig. 1B). Indeed, immediately before cells were replated in 10% FBS (0 h), the majority of the cells were found in the G_0_/G_1_ phases, which was also true for cells analyzed at 12 h of supplementation (Fig. 1B). After 24 h, it was possible to observe progression through S phase, and by 36 and 48 h, NIH 3T3 cells were able to progress through the G_2_ and M phases, and subsequently to G_1_ (Fig. 1B). Western blot analysis identified maximum hyperphosphorylation of Rb protein and increased accumulation of cyclin A at 24 h after supplementation, indicating progression through G_1_ phase and entry into S phase during this period (Fig. 1C). Also, phosphorylation of histone H3, a marker of mitotic progression, was observed after 48 h of supplementation (Fig. 1C). To estimate more precisely the progression of NIH 3T3 cells through the cell cycle in this model, the expression levels of cyclins D2, E2, A2, and B2 were assessed by quantitative PCR (qPCR). Expression peaks were observed for cyclin D2 after 12 h of supplementation, for cyclins E2 and A2 after 24 h, and for cyclin B2 after 36 and 48 h (data not shown), further indicating that synchronized NIH 3T3 cells are able to progress uniformly through the cell cycle upon serum supplementation. Together, these results indicate that our synchronization/release method provides an interesting model for evaluating the regulation of lipid droplets during different cell cycle phases, and for that reason, this method was used throughout this study.

**FIG 2:**
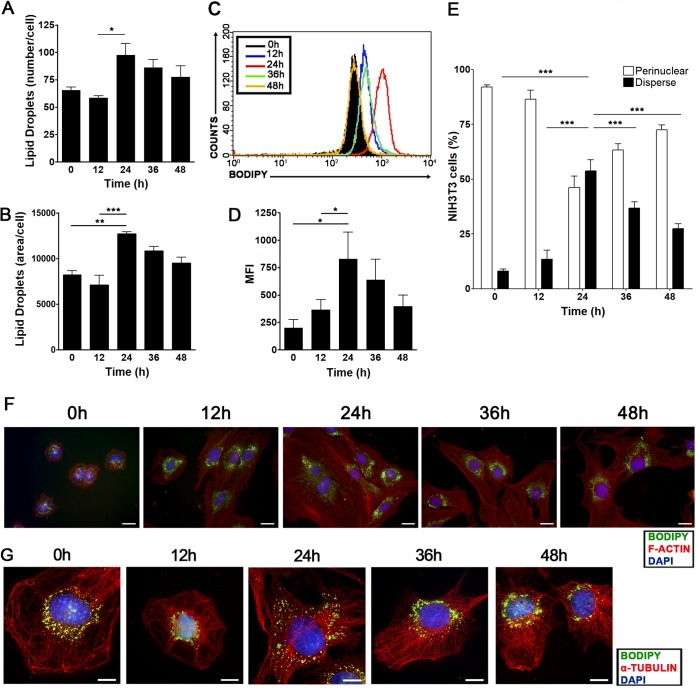
The quantity and subcellular localization of lipid droplets vary during cell cycle progression starting from G_0_/G_1_-phase-synchronized NIH 3T3 cells. NIH 3T3 cells were synchronized by confluence and serum starvation as shown in Fig. 1A and were then replated at a low density and supplemented with a medium containing 10% FBS. (A and B) Lipid droplet quantification through Oil Red O staining. NIH 3T3 cells were fixed on coverslips and were stained with the fluorescent dye Oil Red O at the indicated time points. The number (A) and total area (arbitrary units) (B) of lipid droplets per cell are shown as means ± standard deviations from three independent experiments. Asterisks indicate significant differences in values (*, *P* < 0.05; **, *P* < 0.01; ***, *P* < 0.001). (C and D) Lipid droplet quantification through flow cytometry. NIH 3T3 cells were stained with Bodipy at the indicated time points and were analyzed by flow cytometry. Data are shown in a fluorescence intensity overlay graph (C) or are expressed as the mean fluorescence emission intensity (MFI) (D). (E to G) Subcellular localization of lipid droplets. NIH 3T3 cells were fixed on coverslips at the indicated time points and were treated with a polyclonal anti-α-tubulin antibody for the detection of microtubules or with phalloidin for F-actin visualization (red), with the hydrophobic fluorescent probe Bodipy for lipid droplet staining (green), and with DAPI for nuclear staining (blue). (E) Graph showing the percentage of NIH 3T3 cells displaying perinuclear or dispersed lipid droplets at the indicated time points. Data in bar graphs are means; error bars, standard deviations. (F and G) Fluorescence microscopy analyses show cellular morphology and the subcellular localization of lipid droplets at the indicated time points. Representative images were captured at objective magnifications of ×40 (F) and ×100 (G). Bars, 10 μm. All data are representative of the results of at least three independent experiments.

First, we assessed whether the quantity of lipid droplets varied during the progression of NIH 3T3 cells through the cell cycle. Lipid droplets were quantified by two means: (i) using the lipophilic probe Oil Red O to stain lipid droplets, we counted the droplets (Fig. 2A) and measured their total area per cell (Fig. 2B) on photographed fluorescence images; (ii) using Bodipy staining, we were able to quantify these organelles in a flow cytometer via the mean fluorescence intensity (MFI) displayed by the samples analyzed (Fig. 2C and D). It was observed that both the number and the total area of lipid droplets increased during progression through the cell cycle, peaking at 24 h after serum supplementation and slowly decreasing afterwards (Fig. 2A and B). Analysis by flow cytometry revealed the same pattern in fluorescence images, with a maximum increase in Bodipy fluorescence at 24 h after serum supplementation (Fig. 2C and D), confirming that the quantity of lipid droplets is tightly regulated during cell cycle progression. Using the same model, we also evaluated the subcellular localization of lipid droplets in NIH 3T3 cells by fluorescence microscopy. For this purpose, we marked lipid droplets with Bodipy while staining microtubules to determine the cell boundaries and using 4',6-diamidino-2-phenylindole (DAPI) as a nuclear marker. We observed an increased fraction of cells displaying dispersed localization of lipid droplets through the cytoplasm at 24 h after serum supplementation (Fig. 2E to G), whereas perinuclear localization of lipid droplets was preferentially seen at all other time points evaluated (Fig. 2E to G). Interestingly, the increases in the quantity and dispersed localization of lipid droplets were associated with entry into S phase (Fig. 1B). To further support this idea, we assessed lipid droplet regulation in a different cell type, the nontransformed rat epithelial cell line IEC-6. Using the same synchronization/release protocol as for NIH 3T3 cells, we observed that synchronized IEC-6 cells were able to reenter the cell cycle after serum supplementation in the same way as NIH 3T3 cells (data not shown). Also, IEC-6 cells showed dispersed localization of lipid droplets and a peak increase in Bodipy fluorescence intensity upon S-phase entry, and Oil Red O staining revealed that the number of lipid droplets and their total area per cell increased as IEC-6 cells progressed through the cell cycle (data not shown).

**FIG 3:**
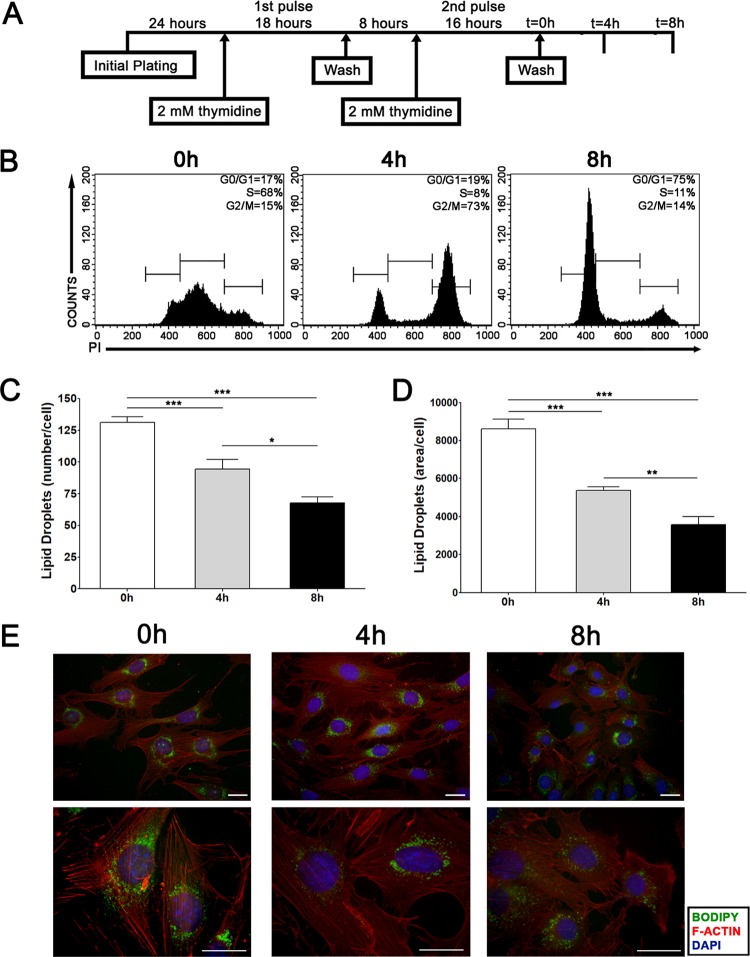
The quantity and subcellular localization of lipid droplets vary during cell cycle progression starting from S-phase-synchronized NIH 3T3 cells. (A) Experimental scheme of NIH 3T3 cell synchronization by a double thymidine block. (B) Cell cycle analysis was performed by propidium iodide staining of NIH 3T3 cells either before thymidine block release (0 h) or 4 h or 8 h after release. The percentage of cells in each stage of the cell cycle (G_0_/G_1_, S, and G_2_/M) is given. (C and D) Lipid droplet quantification through Oil Red O staining. NIH 3T3 cells were fixed on coverslips and were stained with the fluorescent dye Oil Red O at the indicated time points. The number of lipid droplets (C) and their total area per cell (arbitrary units) (D) are shown. Bars represent means from three independent experiments; error bars, standard deviations. Asterisks indicate significant differences in values (*, *P* < 0.05; **, *P* < 0.01; ***, *P* < 0.001). (E) Subcellular localization of lipid droplets. NIH 3T3 cells were fixed on coverslips at the indicated time points and were treated with phalloidin for F-actin visualization (red), with the hydrophobic fluorescent probe Bodipy for lipid droplet staining (green), and with DAPI for nuclear staining (blue). Fluorescence microscopy analysis shows cellular morphology and the subcellular localization of lipid droplets at the indicated time points. Representative images were captured at objective magnifications of ×40 (top) and ×100 (bottom). Bars, 10 μm. All data are representative of the results of at least three independent experiments.

In order to avoid any bias from a cell synchronization method using serum starvation, the potential cell cycle-mediated regulation of lipid droplets was further evaluated in proliferating NIH 3T3 cells subjected to a double thymidine block in order to induce S-phase arrest (Fig. 3A). Following synchronization, cells were analyzed either immediately (0 h) or 4 or 8 h after thymidine release (Fig. 3A). This method was able to arrest NIH 3T3 cells in S phase, as seen through propidium iodide staining (Fig. 3B). Also, the majority of NIH 3T3 cells initially arrested at S phase progressed through G_2_/M after 4 h of release from the thymidine block, reaching the G_1_ phase 8 h after release (Fig. 3B). In accordance with what was observed previously, lipid droplet quantification by Oil Red O staining in this model also revealed an increased number and total area of lipid droplets in NIH 3T3 cells progressing through S phase (Fig. 3C and D). Moreover, analysis of the subcellular localization of lipid droplets revealed that most S-phase cells displayed more dispersion of these organelles through the cytoplasm than cells progressing through the G_2_/M or G_1_ phase of the cell cycle (Fig. 3E). Together, these data show that lipid droplets are tightly regulated organelles during the progression of NIH 3T3 and IEC-6 cells through the cell cycle.

### Lipid droplets display dynamic reorganization of their subcellular localization throughout the progression of NIH 3T3 cells through the cell cycle.

**FIG 4:**
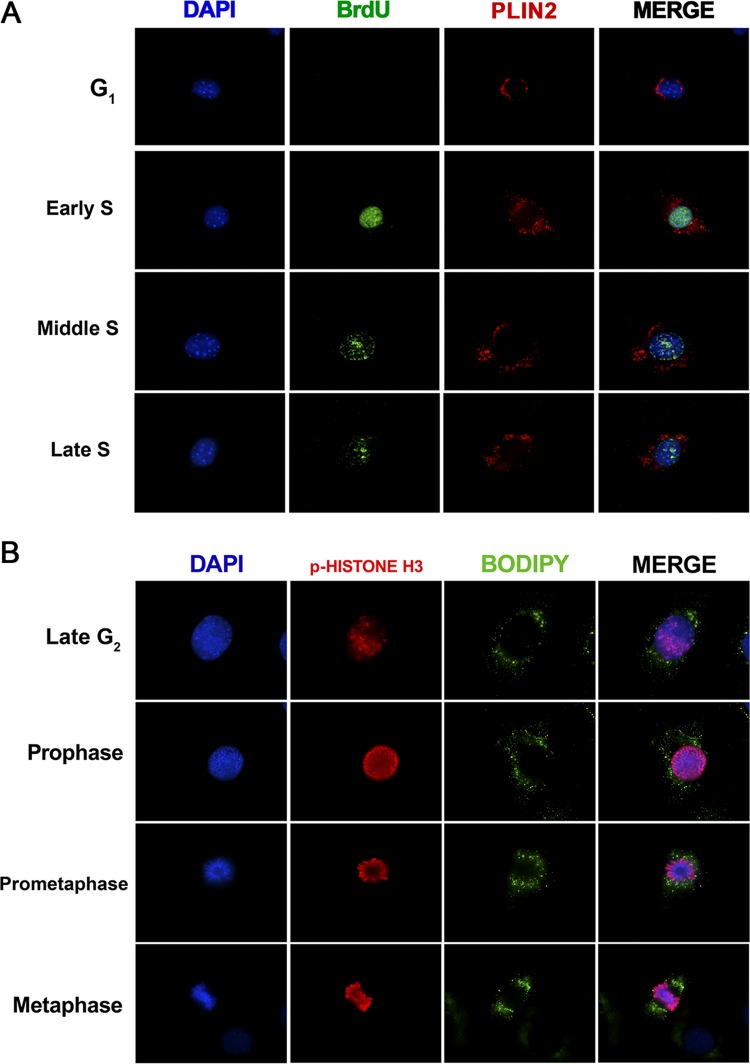
The subcellular localization of lipid droplets is highly dynamic during different cell cycle phases. NIH 3T3 cells were synchronized by confluence and serum starvation as shown in Fig. 1A and were then replated at a low density and supplemented with a medium containing 10% FBS. (A) Synchronized NIH 3T3 cells on coverslips were incubated with 20 μM BrdU for 30 min before fixation at the indicated time points. Later, cells were treated with anti-BrdU for the detection of DNA synthesis (green) and with anti-PLIN2 for lipid body staining (red), along with DAPI for nuclear staining (blue). Fluorescence microscopy analysis shows the subcellular localization of lipid droplets and BrdU nuclear staining at the indicated time points. (B) Synchronized NIH 3T3 cells were fixed on coverslips at 48 h after serum supplementation and were then treated with antibodies to phospho-histone H3 (p-histone H3) for the detection of chromosome condensation (red) and with Bodipy for lipid droplet staining (green), along with DAPI for nuclear staining (blue). Fluorescence microscopy analysis shows the subcellular localization of lipid droplets during G_2_ and mitosis. All data are representative of the results of at least three independent experiments.

In order to perform a more detailed analysis of the subcellular localization of lipid droplets during S phase, NIH 3T3 cells were briefly pulsed with BrdU to determine the stage of DNA synthesis by fluorescence microscopy (44, 45). PLIN2 staining was used to visualize lipid droplets, because PLIN2 is a well-recognized protein marker for these organelles (15, 17). It was noted that while BrdU-negative cells (stained 12 h after supplementation) did not display lipid droplet dispersion, BrdU-positive cells (stained 24 h after supplementation) showed greater dispersion of these organelles through the cytoplasm (Fig. 4A), in agreement with the data presented above showing increased dispersion of lipid droplets in S phase. Moreover, it was possible to observe that cells with homogeneous BrdU staining in the nucleus, indicative of early S phase, were still displaying perinuclear lipid droplets (Fig. 4A). On the other hand, cells with perilaminar and/or perinucleolar BrdU staining, indicative of the middle of S phase, showed the greatest dispersion of lipid droplets (Fig. 4A). Finally, cells with a decreased number of BrdU foci, indicative of the end of S phase, again showed perinuclear localization of lipid droplets (Fig. 4A). These data imply that lipid droplets of NIH 3T3 cells show highly dynamic changes in their localization during S phase, which may be coupled with DNA replication. In an attempt to further characterize the motion of lipid droplets through cell cycle progression, we also analyzed their subcellular localization during late G_2_ and the initial steps of mitosis by using phosphorylated histone H3 as a marker of chromosome condensation (46) (Fig. 4B). Using Bodipy to stain lipid droplets, we observed that these organelles became symmetrically polarized when cells approached metaphase (Fig. 4B), demonstrating the highly dynamic behavior of lipid droplets throughout the NIH 3T3 cell cycle.

### Increased quantity and dispersed localization of lipid droplets are characteristic of NIH 3T3 cells progressing through S phase.

**FIG 5:**
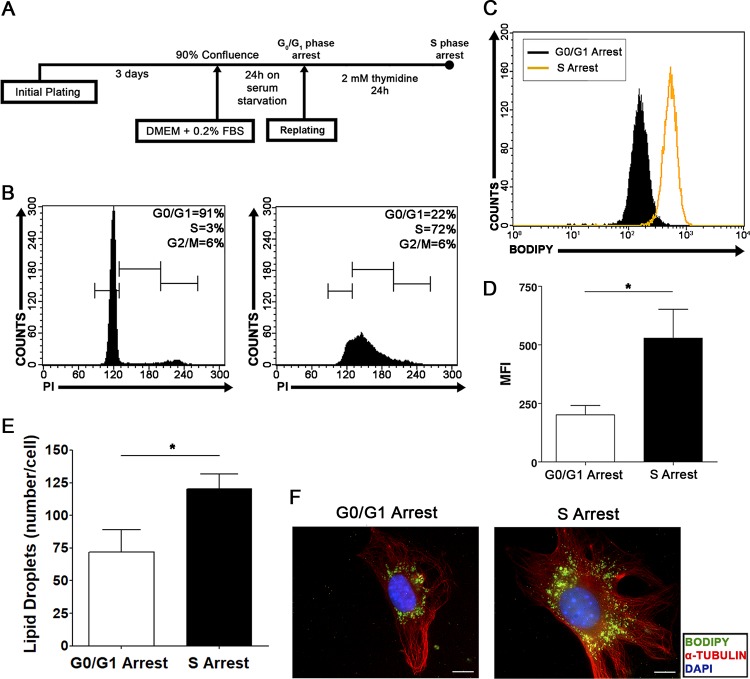
S-phase-arrested NIH 3T3 cells display increased numbers and dispersed localization of lipid droplets. (A) Experimental scheme of NIH 3T3 cell cycle arrest by thymidine administration. (B) Cell cycle analysis was performed on NIH 3T3 cells after synchronization by confluence and serum starvation (left) or after thymidine incubation (right). The percentage of cells in each stage of the cell cycle (G_0_/G_1_, S, and G_2_/M) is shown. (C and D) Lipid droplet quantification through flow cytometry. G_0_/G_1_- or S-phase-arrested NIH 3T3 cells were stained with Bodipy and were analyzed by flow cytometry. Data are shown in a fluorescence intensity overlay graph (C) or are expressed as the mean fluorescence emission intensity (MFI) (D). Data are means; error bars, standard deviations. An asterisk indicates a significant difference in values (*, *P* < 0.05). (E) Lipid droplet quantification by Oil Red O staining. G_0_/G_1_- or S-phase-arrested NIH 3T3 cells were fixed on coverslips and stained with the fluorescent dye Oil Red O. The number of lipid droplets per cell was determined using ImageQuant TL software (GE Healthcare). Data are means ± standard deviations from three independent experiments. An asterisk indicates a significant difference in values (*P* < 0.05). (F) Subcellular localization of lipid droplets. G_0_/G_1_- or S-phase-arrested NIH 3T3 cells were fixed on coverslips and were treated with a polyclonal anti-α-tubulin antibody for the detection of microtubules (red), with the hydrophobic fluorescent probe Bodipy for lipid droplet staining (green), and with DAPI for nuclear staining (blue). Fluorescence microscopy analysis shows cellular morphology and the subcellular localization of lipid droplets in G_0_/G_1_- or S-phase-arrested NIH 3T3 cells. All data are representative of the results of at least three independent experiments.

Intending to confirm our data pointing to specific regulation of lipid droplets during the S phase of the cell cycle, we performed a specific S-phase arrest in NIH 3T3 cells with thymidine incubation. To this end, cells were initially synchronized in the G_0_/G_1_ phases as described above and were released to reenter the cell cycle in the presence of thymidine (Fig. 5A). After 24 h of cell cycle release in the presence of thymidine, cells clearly accumulated in S phase, in contrast to cells arrested in the G_0_/G_1_ phases (Fig. 5B). S-phase-arrested cells were then compared with G_0_/G_1_-phase-arrested cells, and the quantity and subcellular localization of lipid droplets were analyzed as described above. S-phase-arrested cells displayed a higher quantity of lipid droplets than cells arrested in the G_0_/G_1_ phases, as evaluated by Bodipy fluorescence intensity (Fig. 5C and D) and by Oil Red O staining and quantification (Fig. 5E). Moreover, most of the S-phase-arrested cells showed dispersed localization of lipid droplets through the cytoplasm, whereas the majority of cells arrested in the G_0_/G_1_ phases exhibited perinuclear lipid droplets (Fig. 5F). These results support the data presented above (Fig. 2 and 3) and together show that a specific increase in lipid droplets and a specific pattern of subcellular localization occur during the S phase of the cell cycle in NIH 3T3 cells.

### NIH 3T3 cells transformed with the H-*rasV12* oncoprotein present an increased quantity of lipid droplets and accumulation of the PAT protein PLIN2.

**FIG 6:**
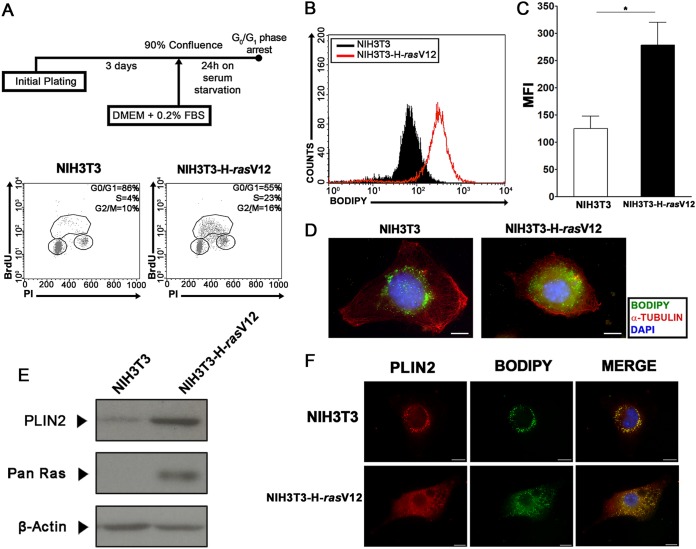
Transformed cells display accumulation of the lipid droplet structural protein PLIN2 and increased numbers of lipid droplets. (A) Experimental scheme of synchronization of NIH 3T3 and NIH 3T3-H-*rasV12* cells. Following a confluence and serum starvation procedure (top), cell cycle analysis was performed on NIH 3T3 (bottom left) or NIH 3T3-H-*rasV12* (bottom right) cells, and the percentage of cells in each stage of the cell cycle (G_0_/G_1_, S, and G_2_/M) is shown. (B and C) Lipid droplet quantification through flow cytometry. NIH 3T3 or NIH 3T3-H-*rasV12* cells were stained with Bodipy after the confluence and serum starvation procedure and were then analyzed by flow cytometry. Data are shown in a fluorescence intensity overlay graph (B) or are expressed as mean fluorescence emission intensity (MFI) (C). Bars indicate means; error bars, standard deviations. An asterisk indicates a significant difference in values (*, *P* < 0.05). (D) NIH 3T3 cells or NIH 3T3-H-*rasV12* cells were fixed on coverslips after confluence and serum starvation and were then treated with a polyclonal anti-α-tubulin antibody for the detection of microtubules (red), with the hydrophobic fluorescent probe Bodipy for lipid droplet staining (green), and with DAPI for nuclear staining (blue). Fluorescence microscopy analysis shows cellular morphology and the accumulation of lipid droplets in NIH 3T3 or NIH 3T3-H-*rasV12* cells. Bars, 10 μm. (E) PLIN2 accumulation in NIH 3T3 or NIH 3T3-H-*rasV12* cells. Western blotting was performed using antibodies to the indicated proteins after the confluence and serum starvation procedure. (F) NIH 3T3 or NIH 3T3-H-*rasV12* cells were treated with a polyclonal anti-PLIN2 (red) antibody, with the hydrophobic fluorescent probe Bodipy for lipid droplet staining (green), and with DAPI for nuclear staining (blue). Bars, 10 μm. All data are representative of the results of at least three independent experiments.

In order to characterize the differences in the regulation of lipid droplets in cells with an uncontrolled cell cycle, NIH 3T3 cells were transformed with the constitutively active H-*rasV12* oncoprotein (47). As reported previously, NIH 3T3-H-*rasV12* cells display features of transformation *in vitro* (47). Both NIH 3T3 and NIH 3T3-H-*rasV12* cells were grown to confluence and subjected to serum starvation for cell cycle synchronization as shown in Fig. 6A. Propidium iodide and BrdU analysis showed that this procedure was able to induce G_0_/G_1_ arrest in wild-type NIH 3T3 cells but failed to induce the same effect in NIH 3T3-H-*rasV12* cells (Fig. 6A). According to our previously published data, epithelial cells displayed an increased quantity of lipid droplets after transformation (32). Therefore, we assessed the quantity of lipid droplets in wild-type NIH 3T3 cells and NIH 3T3-H-*rasV12* cells after confluence and serum starvation. We observed a remarkable increase in Bodipy fluorescence intensity in transformed NIH 3T3-H-*rasV12* cells (Fig. 6B and C), which was then confirmed by fluorescence microscopy analysis (Fig. 6D). These data suggest the existence of differential regulation of the lipid droplet quantity in transformed cells *in vitro*. It has been reported that PLIN2, one of the members of the PAT family of lipid droplet structural proteins, is important for intracellular lipid accumulation, and its participation in lipid droplet biogenesis has been suggested (15, 16, 18). Based on this, an increase in PLIN2 levels in NIH 3T3-H-*rasV12* cells could be correlated with the increased quantity of lipid droplets observed in these cells. Thus, we evaluated PLIN2 levels and subcellular localization in wild-type NIH 3T3 cells and NIH 3T3-H-*rasV12* cells. Western blot assays revealed higher PLIN2 levels in NIH 3T3-H-*rasV12* cells than in NIH 3T3 cells (Fig. 6E). Also, we observed the colocalization of PLIN2 staining with Bodipy fluorescence by fluorescence microscopy (Fig. 6F).

### PLIN2 is not an oncoprotein in NIH 3T3 cells.

**FIG 7:**
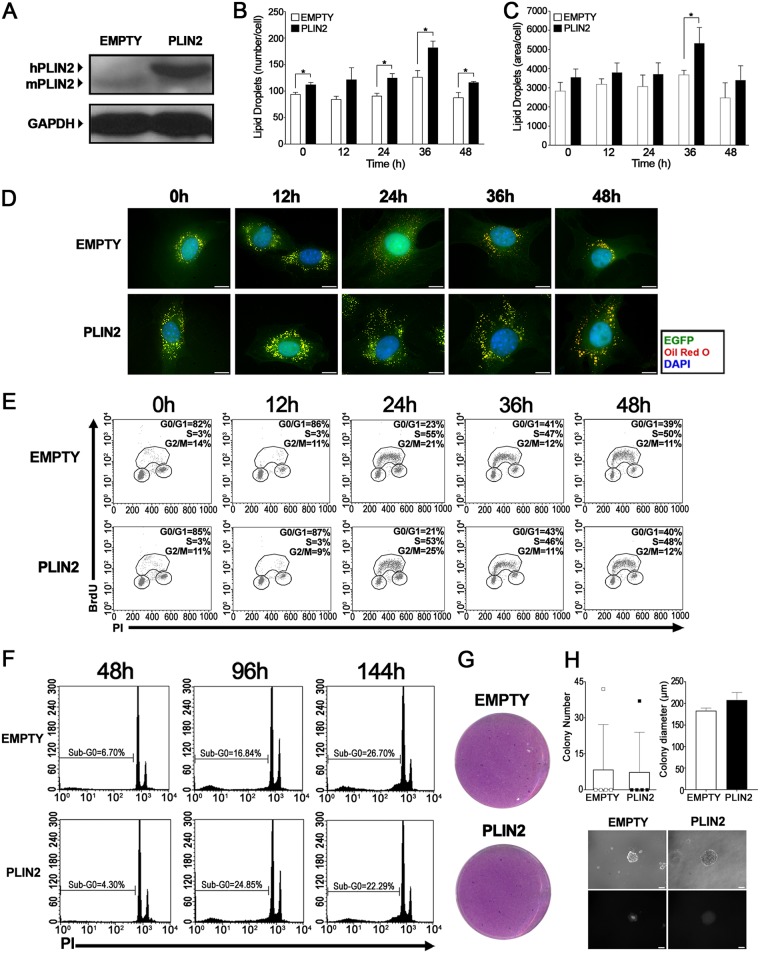
NIH 3T3 cells with PLIN2 overexpression do not show altered cell proliferation or display a transformation phenotype *in vitro*. (A) PLIN2 accumulation in transduced NIH 3T3 cells. Whole-cell extracts were prepared 24 h after supplementation and replating of synchronized cells transduced with an empty vector or a *PLIN2* expression vector. The extracts were later separated by SDS-PAGE. Western blotting was performed using antibodies to the indicated proteins. GAPDH, glyceraldehyde 3-phosphate dehydrogenase. (B and C) Lipid droplet quantification through Oil Red O staining. After supplementation, transduced NIH 3T3 cells were fixed on coverslips and stained with Oil Red O at the indicated time points. The number (B) and total area (arbitrary units) (C) of lipid droplets per cell were determined using ImageQuant TL software (GE Healthcare). Results are means; error bars, standard deviations. An asterisk indicates a significant difference between values (*, *P* < 0.05). (D) Subcellular localization of lipid droplets. After supplementation, transduced NIH 3T3 cells were fixed on coverslips at the indicated time points and were treated with Oil Red O for lipid droplet staining (red) and with DAPI for nuclear staining (blue). Cell morphology was observed through EGFP fluorescence (green). Fluorescence microscopy analysis shows the subcellular localization of lipid droplets in transduced cells. Bar, 10 μm. (E) Cell cycle progression assay. After synchronization, transduced NIH 3T3 cells were replated at a low density, supplemented with a medium containing 10% FBS, and incubated with 20 μM BrdU for 30 min before fixation at the indicated time points. Later, cell cycle analysis was performed using an FITC-conjugated anti-BrdU antibody together with propidium iodide staining. (F) Cell death assay. After synchronization, transduced NIH 3T3 cells were replated at a low density and were supplemented with a medium containing 0.2% FBS. Sub-G_0_ DNA content was evaluated with propidium iodide (PI) staining at the times indicated. (G) Focus formation assay. Following transduction, cells were mixed 1:5 with wild-type NIH 3T3 cells. After 12 days, cells were visualized by crystal violet staining. (H) Semisolid-medium growth assay. Following transduction, NIH 3T3 cells were cultured in DMEM containing 0.6% agarose. After 20 days, colonies were counted (top left), and representative colonies were measured (top right) and visualized by both phase-contrast (center) and fluorescence (bottom) microscopy for the detection of EGFP expression. All data are representative of the results of at least three independent experiments.

In our efforts to understand the role of lipid droplet biogenesis in cell cycle regulation, the next step was to induce the accumulation of PLIN2 in NIH 3T3 cells through stable overexpression of the *PLIN2* gene. For this purpose, NIH 3T3 cells were transduced with a retroviral vector containing the human *PLIN2* gene under the control of the cytomegalovirus promoter. Transduction efficiency was determined by flow cytometry using the enhanced green fluorescent protein (EGFP) reporter as a reference (data not shown). The transduced cells were then grown to confluence and subjected to serum starvation; the cells were replated at a low density with serum supplementation; and the quantity and subcellular localization of lipid droplets were determined. High levels of human PLIN2 protein (hPLIN2) were confirmed by Western blotting at 5 days after transduction (24 h after replating with serum supplementation) (Fig. 7A). Through Oil Red O staining, we observed higher numbers of lipid droplets in cells overexpressing *PLIN2* than in control cells (Fig. 7B), together with an increase in their total area per cell after 36 h of serum supplementation (Fig. 7C). This was also reflected upon fluorescence microscopy analysis of lipid droplets, where we observed not only an increase in the numbers of lipid droplets in cells overexpressing *PLIN2* but also increased dispersion of lipid droplets through the cytoplasm at most of the time points evaluated (Fig. 7D). In contrast, cells transduced with the control vector exhibited largely dispersed lipid droplets only at 24 h after cell cycle reentry (Fig. 7D), a finding in agreement with the results obtained in our synchronization model above (Fig. 2). These data indicate that increased production of human PLIN2 protein alters the regulation of lipid droplets in NIH 3T3 cells throughout the cell cycle.

Based on the results presented above, we performed a series of assays to evaluate the effect of *PLIN2* overexpression on the proliferation and transformation of NIH 3T3 cells *in vitro*. Still under the synchronization model of confluence and serum starvation, transduced cells were supplemented with a medium containing either 0.2% or 10% serum after cell cycle release in order to evaluate if *PLIN2* overexpression could induce a hyperproliferative phenotype in NIH 3T3 cells. Crystal violet analysis revealed no differences in cell accumulation between control cells and cells overexpressing the *PLIN2* gene, independently of serum concentration (data not shown). In the same manner, BrdU incorporation analysis failed to detect differences in cell cycle progression between control NIH 3T3 cells and NIH 3T3 cells with high levels of PLIN2 protein (Fig. 7E), thus implying that *PLIN2* overexpression is not able to induce proliferation. Still attempting to determine if the *PLIN2* gene is able to induce other transformation hallmarks in NIH 3T3 cells, we performed a cell death assay in synchronized cells grown under conditions of serum withdrawal. By sub-G_0_ content analysis, we observed no differences between control cells and cells overexpressing the *PLIN2* gene at the time points analyzed (Fig. 7F). Finally, *in vitro* cellular transformation assays were conducted in transduced NIH 3T3 cells to evaluate the carcinogenic potential of PLIN2. First, neither control cells nor *PLIN2*-overexpressing cells induced focus formation when analyzed by crystal violet staining, suggesting that none of these constructs are able to disrupt cell-cell contact inhibition (Fig. 7G). Second, transduced NIH 3T3 cells were plated on a semisolid agarose medium, which prevents cells from adhering to a solid substrate. Whereas the vast majority of colonies appearing under both conditions were fluorescent, and thus were likely formed by infected NIH 3T3 cells, we could not find differences in quantity, diameter, or morphology between colonies of control cells and cells with increased *PLIN2* expression (Fig. 7H). Taken together, these results clearly indicate that *PLIN2* overexpression is unable to induce a transformed phenotype in a noncancerous cell line, thus discarding the hypothesis that this gene may have an oncogene function in NIH 3T3 cells.

### PLIN2 accumulation is associated with highly proliferative human colon adenocarcinoma tissues.

**FIG 8:**
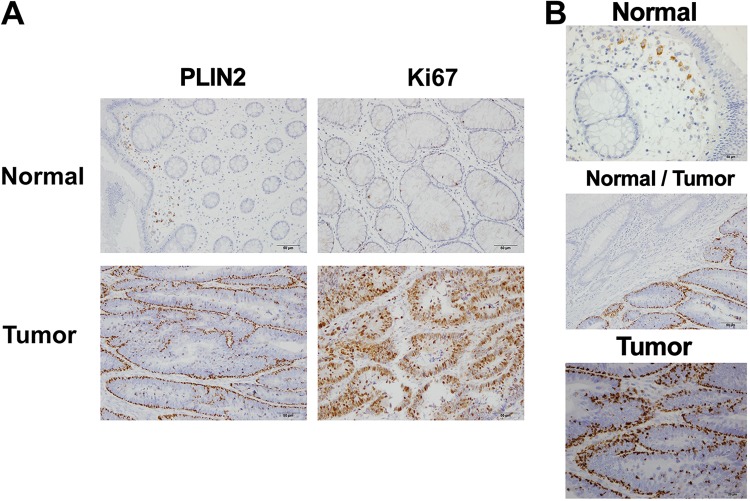
Staining for PLIN2 protein is specific for colon adenocarcinoma tissue. Human colon adenocarcinoma tissues from patients were evaluated for PLIN2 protein accumulation by immunohistochemistry. (A) Bright-field microscopy images from representative samples for PLIN2 and Ki-67 staining showing colon adenocarcinoma tumoral tissue (bottom) or adjacent normal tissue from the same sample (top). (B) Representative images for PLIN2 staining of the neighboring region between colon adenocarcinoma and normal tissues (center) and details of normal tissue (top) and tumor tissue (bottom). All results are representative of samples from three patients.

As discussed previously, some neoplastic cells display increased quantities of lipid droplets (32), a finding also confirmed by our present data (Fig. 5). These observations draw attention to the use of these organelles, or their associated proteins, as biomarkers for some cancer types. Following this reasoning, we evaluated the presence and pattern of PLIN2 protein by immunohistochemistry, as a marker of lipid droplet accumulation in samples of human colon adenocarcinoma tissues and in paired adjacent normal tissues. Patient samples were processed as described in Materials and Methods, and immunohistochemical images are presented in Fig. 8. In this analysis, it is possible to observe a remarkable difference between the PLIN2 staining pattern in tumor tissue, which displays a considerably higher accumulation of PLIN2, and that in paired adjacent normal tissue (Fig. 8A). Interestingly, this increase in PLIN2 positivity is coupled with an increase in the intensity of Ki-67 staining (Fig. 8A), correlating PLIN2 accumulation with enhanced cell proliferation. Moreover, at a higher magnification, we observed that the punctate PLIN2 staining in the normal tissue (Fig. 8B, top) localizes at infiltrating cells such as macrophages, whereas the great majority of PLIN2 staining in tumor tissue localizes specifically at the cancerous epithelial cells (Fig. 8B, bottom). Finally, by analyzing the border region between the colon tumor and the normal tissue, it is possible to clearly point out the significant difference in PLIN2 positivity observed in the tumor tissue (Fig. 8B, center). Collectively, these data highlight PLIN2 protein as a potential biomarker for highly proliferative human colon adenocarcinoma.

## DISCUSSION

In the present study, we demonstrated that lipid droplets are highly dynamic organelles throughout the cell cycle progression of two nontransformed mammalian cell lines, NIH 3T3 murine fibroblasts and IEC-6 rat intestinal epithelial cells. Using a reliable model to analyze the variations of a specific organelle during different cell cycle phases (Fig. 1), we were able to determine that these cells display an increased number of lipid droplets and higher dispersion of these organelles through the cytoplasm upon entering the S phase (Fig. 2, 3, and 5). Remarkably, we observed that full dispersion of lipid droplets in NIH 3T3 cells takes place mostly at the middle of S phase (Fig. 4), and in-depth analysis of the distribution of lipid droplets during mitosis revealed their polarization before cell division (Fig. 4). Although other studies have already linked lipid droplet regulation with the cell cycle in yeast cells (48, 49), this work reveals for the first time the dynamic behavior of lipid droplets through the different cell cycle phases in mammalian cells.

Our group has already observed a positive correlation between an increase in the number of lipid droplets and cell proliferation in colon cancer cells (32). Also, lipid metabolism is considered pivotal to supporting such increased proliferation with materials and energy (50). Accordingly, a loss of lipid droplet-targeted lipolysis combined with inhibition of fatty acid production in budding yeast leads to the complete abolishment of yeast cell cycle progression by G_1_ arrest (41). Similarly, impairment of lipid droplet biogenesis in a caveolin-1 knockout mouse model correlated with G_1_ cell cycle arrest in hepatocytes after partial hepatectomy (40). These data suggest that cell cycle progression and lipid homeostasis are coordinated by a shared mechanism acting at the G_1_/S transition, thus implying that lipid droplet maintenance, biogenesis, or consumption is involved in cell cycle progression through S phase. In fact, a lipid-dependent metabolic checkpoint was recently described in late G_1_ phase (51). Moreover, the fast lipid droplet dispersion within S phase identified in our study through BrdU staining is correlated with different stages of DNA synthesis, suggesting that molecules involved in the G_1_/S transition or S-phase progression could be acting in this process. One possible reason for this dynamic movement is to increase the interaction of lipid droplets with organelles such as mitochondria (52) and peroxisomes (53) and thus promote fatty acid β-oxidation to energetically fuel cell proliferation or to provide building blocks for cell growth (54, 55). Microtubules seem to be directly involved with lipid droplet dynamics (56, 57), and microtubule-dependent dispersion of lipid droplets has recently been described as a response to nutrient starvation, increasing fatty acid β-oxidation by directing interactions between these organelles and mitochondria (58). Lipid droplets might also act as transporters of hydrophobic proteins, since these organelles have been described as protein sequestration sites in several models (59). Transport of free histones coupled with DNA synthesis is an interesting possibility, based on a previous report describing the storage and release of histones in lipid droplets during *Drosophila* development to form new nucleosomes for the initial rounds of DNA replication in the early embryo syncytium (60). Finally, recent work describing differences in the production and localization of specific lipid species between interphase and cytokinesis (61), with involvement of lipid droplet-resident enzymes, reinforces the importance of these organelles as sources of important lipid species for different cell cycle processes.

Increasing evidence about lipid droplet accumulation in cancers suggests these organelles as possible targets for cancer therapy. Following this idea, this work revealed that NIH 3T3 cells transformed with the constitutively active oncoprotein H-*rasV12* exhibit a higher number of lipid droplets than their wild-type counterparts, together with increased accumulation of the PAT family structural protein PLIN2 (Fig. 6). The observation that these transformed cells displayed a dramatic increase in the quantity of lipid droplets draws attention to the enhanced lipogenesis observed in several cancer types. This could potentially enhance the signaling pathways involved in transformation or could metabolically sustain tumor growth and consequently give cancer cells a proliferative advantage. Indeed, the study previously conducted by our group showed the same effect in IEC-6 cells transformed with H-*rasV12*, where an increase in lipid droplet numbers was directly implicated in the production of inflammatory mediators, enhancing their proliferation rate via autocrine signaling (32). Additionally, PLIN2 has been identified as a key component for lipid droplet biogenesis (18), and its accumulation is presumably responsible for the increased intracellular lipid content in these transformed cells. Based on this, we used retroviral vectors to induce overexpression of the *PLIN2* gene in NIH 3T3 cells, which greatly increased the numbers of lipid droplets and augmented their dispersion in our model of cell cycle synchronization and release (Fig. 7). Also, these findings are in accordance with the data concerning the lipid droplet behavior presented by NIH 3T3-H-*rasV12* cells. However, *PLIN2* overexpression by itself could not promote cell proliferation or induce a transformed phenotype in NIH 3T3 cells (Fig. 7). These data demonstrate that PLIN2 does not possess the characteristics of an oncoprotein in NIH 3T3 cells and suggest that the increase in intracellular lipid content alone does not have a great impact on initial tumorigenesis.

It is noteworthy that during the present study, attempts to reduce lipid droplet biogenesis in NIH 3T3 cells were made through both pharmacological inhibition of the cytosolic phospholipase A_2_ (cPLA_2_) enzyme and repression of the endogenous *Plin2* gene by a short hairpin RNA (shRNA) approach. Whereas cPLA_2_ inhibition was able to reduce PGE_2_ production, it failed to reduce the number and total area of lipid droplets in these cells (data not shown). Also, while stable expression of a *Plin2*-targeted shRNA successfully reduced endogenous PLIN2 protein levels, this reduction was not enough to reduce lipid droplet biogenesis or to significantly alter cell cycle progression from that in NIH 3T3 cells transduced with an irrelevant shRNA gene (data not shown). It has been shown previously that inhibition of *Plin2* expression alone in murine fibroblasts is not enough to reduce lipid droplet numbers, due to functional compensation from another PAT protein, PLIN3 (62), and inhibition of both genes is necessary to actually repress lipid droplet biogenesis in these cells (62). On the other hand, it is notable that *PLIN2*-overexpressing NIH 3T3 cells and NIH 3T3-H-*rasV12* cells display such distinct proliferative behaviors while equally showing increased lipid droplet accumulation. An in-depth evaluation of lipid droplet protein and lipid contents comparing these two cell lines may reveal interesting targets for further studies. Furthermore, the data presented in this work do not exclude a role for lipid droplets in well-established tumors, which depend on important modifications in energy metabolism (63, 64), on a permissive microenvironment for the maintenance or invasion of tumor cells (65), or on conditions that enable cells to withstand extreme situations, such as hypoxia (66). Among these characteristics, necessary for the most aggressive malignant cells, differential regulation of lipid metabolism could directly impact tumor development. Nevertheless, PLIN2 appears to be an interesting biomarker for highly proliferative colon adenocarcinoma, distinguishing cancerous tissue from normal tissue in paired samples from the same patient to an exceptional degree (Fig. 8). Analysis of PAT protein expression levels has been already performed on neoplastic tissues that display a certain degree of steatosis, revealing that different cancer types display distinct expression patterns of PAT family members (67). Indeed, PLIN2 is the member that attracts the most attention as a potential biomarker, since its differential expression has been observed in several human carcinomas beyond the present work (68–73), although the prognostic significance of PLIN2 expression in colorectal cancer remains to be determined.

In summary, this work describes differential regulation of lipid droplets throughout cell cycle progression in mammalian cells, which is disrupted upon cellular transformation. Also, it was observed that intracellular lipid accumulation *per se*, mediated through PLIN2 accumulation, is not enough to drive cellular transformation. Still, this work identifies an interesting association between lipid droplet accumulation and cell proliferation, as observed during S-phase entry, in oncogenic transformed NIH 3T3-H-*rasV12* cells, or in highly proliferative, Ki-67-positive colon cancer tissue. These results suggest, therefore, the existence of a mechanism that connects cell cycle progression and cell proliferation with lipid accumulation, taking a first step toward understanding the real cause-consequence relationship of the increased lipogenesis present in some cancer cells. We also discuss the possible mechanisms by which lipid droplets may be promoting tumorigenesis, such as the availability of lipids for structural or energetic needs, as well as for the production of inflammatory mediators. Each one of these factors can have an impact on cell proliferation, differentiation, and motility, which are crucial for cancer establishment (74). We also suggest the use of lipid droplets and their associated structural protein PLIN2 as tumor biomarkers for colon adenocarcinoma. In conclusion, better comprehension of the involvement of lipid droplets in tumorigenesis can contribute to the development of new strategies for cancer detection, therapy, and control.

## MATERIALS AND METHODS

### Human subjects and cell culture.

Samples of colon adenocarcinoma tissue and adjacent normal-appearing tissue were collected previously from three patients at the Brazilian National Cancer Institute (INCA). All samples were already fixed in formalin and embedded in paraffin for later utilization. This study was carried out with the approval of the Brazilian National Cancer Institute’s Ethics Committee. NIH 3T3 cells or NIH 3T3-H-*rasV12* cells (47) were cultured in Dulbecco’s modified Eagle’s medium (DMEM) supplemented with either 10% or 0.2% fetal bovine serum (FBS) as indicated in the figure legends, plus 2 mM l-glutamine, penicillin-streptomycin, essential and nonessential amino acids, sodium pyruvate, vitamins, 10 mM HEPES, and 2-mercaptoethanol. NIH 3T3-H-*rasV12* cells were maintained under selective pressure with 7.5 μM puromycin, except when used in experiments. All cell lineages were maintained under 5% CO_2_ at 37°C.

### Plasmid construction.

The retroviral vectors pLIRES2-EGFP (75) and pLIRES2-EGFP-ADRP were used for *PLIN2* overexpression assays. *PLIN2* cDNA was derived from the pLA4 expression vector (17) and was inserted into the pLIRES2-EGFP retroviral backbone plasmid using unique XhoI and SalI restriction sites to create pLIRES2-EGFP-ADRP. To excise the EGFP gene sequence for BrdU incorporation assays, both plasmids were treated with ApaI and NotI, followed by filling using the Klenow DNA polymerase fragment, giving rise to the pL0 and pLADRP plasmids. All vectors were amplified in bacteria and were extracted using the MaxSpeed Plasmid Maxi kit (Qiagen).

### Production of recombinant retrovirus and infection of NIH 3T3 cells.

The ecotropic packing cell lineage BD EcoPack2 (BD Biosciences) was transiently transfected with the retroviral vector pLIRES2-EGFP, pL0, pLIRES2-EGFP-ADRP, or pLADRP by calcium phosphate precipitation for 24 h. The virus-containing cell supernatant was collected 48 h after transfection, mixed 1:1 with fresh medium, supplemented with 8 μg/ml Polybrene (Fluka Chemie, Buchs, Switzerland), and then immediately used for spin infection of 2.5 × 10^4^ NIH 3T3 cells (twice for 45 min each time, at 400 × *g* and room temperature). Infected cells were incubated at 37°C for an additional 24 h, and transduction efficiency was evaluated by green fluorescent protein expression via flow cytometric analysis (pLIRES2-EGFP and pLIRES2-EGFP-ADRP) or by PLIN2 accumulation via Western blot assay (pL0 and pLADRP). To ensure reproducibility, each experiment using the vectors mentioned above was repeated using cells derived from independent viral infections.

### Cell cycle synchronization.

NIH 3T3 cells or NIH 3T3-H-*rasV12* cells were maintained by a combination of confluence and serum starvation for synchronization in the G_1_ phase of the cell cycle. Briefly, 5 × 10^5^ cells were cultured in 75-cm^2^ cell culture flasks for 72 h to reach 90% confluence; then they were washed and maintained for 24 h with a medium supplemented with 0.2% FBS to induce G_1_-phase arrest. Alternatively, 4 × 10^5^ G_1_-arrested NIH 3T3 cells were cultured in 75-cm^2^ cell culture flasks supplemented with a medium containing 10% FBS and 2 mM thymidine (Calbiochem) and were analyzed after 24 h for S-phase arrest. Cells infected with retroviral particles for *PLIN2* overexpression were synchronized by culturing 2.5 × 10^5^ cells in 6-well tissue culture plates for 48 h to reach 90% confluence; then we proceeded as described above for G_1_-phase arrest. For NIH 3T3 cells subjected to a double thymidine block, 7 × 10^4^ cells were cultured in 100-mm culture dishes for 24 h and were then maintained for 18 h with a medium containing 10% FBS and 2 mM thymidine. At the end of the first pulse, cells were washed three times with a medium containing 10% FBS in order to carefully remove all thymidine from the cell culture; then they were cultured with a fresh medium for 8 h. Later, cells were once again maintained with a medium containing 10% FBS and 2 mM thymidine for 16 h to finally achieve S-phase arrest. The same procedure was performed with NIH 3T3 cells plated in gelatin-treated glass coverslips at an initial density of 3 × 10^3^ cells.

### Cell cycle progression analysis.

NIH 3T3 and NIH 3T3-H-*rasV12* cells were maintained by a combination of confluence and serum starvation, then supplemented with a medium containing 10% FBS, and cultured until adhesion (0 h). NIH 3T3 cells were also analyzed at the times indicated in the figures (12, 24, 36, or 48 h). Thirty minutes before fixation, these cells were incubated with 20 nM 5-bromo-2'-deoxyuridine (BrdU) in fresh medium; then they were washed, counted, and fixed in 100% cold ethanol (−20°C) overnight. A total of 5 × 10^5^ cells were then washed in 1% bovine serum albumin (BSA) in a phosphate-buffered saline (PBS) solution, and we proceeded to the DNA denaturation step (2 N HCl and 0.5% Triton X-100; 30 min), followed by inactivation with 0.1 M sodium tetraborate. The cells were then incubated with a fluorescein isothiocyanate (FITC)-conjugated anti-BrdU monoclonal antibody (Phoenix Flow Systems, San Diego, CA) in 1% BSA and 0.5% Tween 20 for 1 h in the dark, followed by incubation with a propidium iodide solution (0.02 mg/ml in PBS, 0.1% Triton X-100, 0.5 mg/ml RNase A) for 24 h at 4°C. A total of 10,000 events were acquired for cell cycle analysis with a FACSCalibur flow cytometer using CellQuest software (BD Biosciences). Experiments with NIH 3T3 cells infected with retroviral particles derived from the pL0 or pLADRP vector were also performed as described above. Alternatively, NIH 3T3 cells synchronized with thymidine were detached by trypsin digestion, washed with PBS, and either stained with an NP-40–propidium iodide solution (75 μM) for 30 min at room temperature or fixed in 100% cold ethanol (−20°C) overnight, followed by incubation with a propidium iodide solution for 24 h at 4°C. DNA content analysis was performed by flow cytometry as described above.

### Analysis of sub-G_0_ DNA content.

G_1_-phase-arrested NIH 3T3 cells infected with pLIRES2-EGFP or pLIRES2-EGFP-ADRP retroviral particles were supplemented with fresh medium containing 0.2% FBS and were seeded at a density of 5 × 10^4^ cells in 6-well tissue culture plates. At the times indicated in the figures, cells were trypsinized and were stained with an NP-40–propidium iodide solution (75 μM). DNA content analysis was performed by collecting 20,000 events for sub-G_0_ DNA using a FACSCalibur flow cytometer and CellQuest software (BD Biosciences).

### Western blotting.

NIH 3T3 cells, NIH 3T3-H-*rasV12* cells, and NIH 3T3 cells infected with pLIRES2-EGFP or pLIRES2-EGFP-ADRP retroviral particles were synchronized by a combination of confluence and serum starvation, and whole-cell extracts were prepared at the times or under the conditions indicated in the figure legends. Briefly, 5 × 10^5^ cells were resuspended in a buffer solution (10 mM EDTA, 40 mM Tris-Cl [pH 7.5], and 60 mM sodium pyrophosphate), followed by 1:1 addition of a 10% SDS solution for cell lysis. The lysates were then incubated at 100°C for 20 min and were mixed 1:5 with loading buffer before samples were applied to cast polyacrylamide gels for SDS-PAGE protein separation, followed by transfer to nitrocellulose membranes using the Trans-Blot Semi-Dry transfer cell system (Bio-Rad). All membranes were blocked with 5% nonfat milk and were incubated overnight with the polyclonal anti-Rb (851) (76), polyclonal anti-PLIN2 (Proteintech North America), or monoclonal anti-pan Ras (F132) (Santa Cruz Biotechnology) primary antibody or for 2 h with the polyclonal anti-cyclin A (C-19; Santa Cruz Biotechnology), polyclonal anti-histone H3 (Cell Signaling), monoclonal anti-phospho-histone H3 (Ser10) (6G3; Cell Signaling), or polyclonal anti-β-actin (Invitrogen) primary antibody, in 1× Tris-buffered saline (TBS) with 0.05% Tween 20 (TBS-T). Immunodetection was performed using the ECL Western blotting analysis system (GE Healthcare).

### Lipid droplet staining for organelle quantification.

NIH 3T3 or NIH 3T3-H-*rasV12* cells were maintained by a combination of confluence and serum starvation, then supplemented with a medium containing 10% FBS, and cultured until adhesion (0 h; for both NIH 3T3 and NIH 3T3-H-*rasV12* cells) or until the times indicated in the figures (12, 24, 36, or 48 h; for NIH 3T3 cells only). A total of 5 × 10^5^ cells were treated with 6 μg/ml Bodipy 493/503 for 10 min at room temperature, washed, and analyzed by flow cytometry for measurement of the mean fluorescence intensity. Alternatively, G_1_-arrested NIH 3T3 cells were plated in gelatin-treated glass coverslips after supplementation with fresh medium containing 10% FBS until adhesion (0 h), until the times indicated in the figures (12, 24, 36, or 48 h), or until synchronization with thymidine (24 h). Alternatively, NIH 3T3 cells were plated in gelatin-treated glass coverslips before being subjected to a double thymidine block and were maintained until release (0 h), or until the times indicated in the figures (4 and 8 h). The cells were then fixed with 2% paraformaldehyde (PFA), treated with 100% propylene glycol (PG), stained for 10 min with 0.5% Oil Red O (in PG), and incubated with 85% PG for 5 min. The coverslips were then washed and visualized by fluorescence microscopy, and 50 cells were chosen randomly from five different coverslip fields for lipid droplet quantification. Images of each cell were taken using the same exposure time, and the number and total area per cell of Oil Red O-stained lipid droplets were quantified using the colony counter module from ImageQuant TL software (GE Healthcare), with the following parameters for lipid droplet detection: sensitivity, 9,500; operator size, 51; noise, 1; background, 1,000; automatic splitting, 9. Lipid droplets in NIH 3T3 cells infected with retroviral particles for *PLIN2* overexpression were also quantified by the same procedure.

### Lipid droplet staining and subcellular localization by fluorescence microscopy.

NIH 3T3 cells, NIH 3T3-H-*rasV12* cells, or NIH 3T3 cells infected with pLIRES2-EGFP or pLIRES2-EGFP-ADRP retroviral particles were synchronized by a combination of confluence and serum starvation, supplemented with fresh medium containing 10% FBS, and plated in gelatin-treated glass coverslips until the times or conditions indicated in the figure legends. NIH 3T3 and NIH 3T3-H-*rasV12* cells were fixed with 2% PFA with 4% saccharose, stained with an anti-α-tubulin antibody and Bodipy, and incubated with 50 nM ammonium chloride. The cells were then permeabilized with 0.05% saponin in PBS and were blocked with 0.2% gelatin and 0.05% saponin before being incubated overnight with an anti-α-tubulin antibody (ICN Biochemicals) in blocking solution, followed by treatment with 0.2% Triton X-100 and Bodipy staining (6 μg/ml). Alternatively, NIH 3T3 cells were fixed with 2% PFA, permeabilized with 1% BSA and 0.5% Tween 20 in PBS for 30 min, and incubated first with 0.165 μM Alexa Fluor 594-phalloidin (Thermo Fisher Scientific) diluted in PBS with 1% BSA for 1 h at room temperature and then with Bodipy stain. The same procedure for F-actin staining was performed in double thymidine block experiments. For PLIN2 and Bodipy staining in NIH 3T3 and NIH 3T3-H-*rasV12*, cells were fixed in 2% PFA, permeabilized in 0.2% Triton X-100, and blocked with 5% FBS and 0.2% Triton X-100 in PBS. Overnight incubation with an anti-PLIN2 antibody (Fitzgerald Industries International) in blocking solution was followed by Bodipy staining. For BrdU and PLIN2 staining, NIH 3T3 cells were incubated with 20 nM BrdU 30 min before fixation with 2% PFA and were then treated with 2 N HCl for DNA denaturation. Then the cells were permeabilized with 0.2% Triton X-100 and were blocked with 1% BSA and 0.5% Tween 20 in PBS before overnight incubation with an FITC-conjugated anti-BrdU monoclonal antibody (Phoenix Flow Systems). The coverslips were then treated with 2% PFA to retain the previously incubated antibodies, followed by PLIN2 immunostaining as described above. For phospho-histone H3 and Bodipy staining, NIH 3T3 cells were fixed with 2% PFA, permeabilized with 0.2% Triton X-100, and blocked with 1% BSA and 0.2% Triton X-100 before being incubated with anti-phospho-histone H3 overnight in blocking solution. Then the cells were stained with Bodipy. Finally, NIH 3T3 cells infected with pLIRES2-EGFP or pLIRES2-EGFP-ADRP retroviral particles were fixed with 2% PFA and were stained with Oil Red O as described above for lipid droplet visualization. Nuclei were stained with 1 μM DAPI in all experiments. Coverslips were then mounted on glass slides using Vectashield antifade mounting medium and were visualized in an Olympus BX60 fluorescence microscope (Olympus). For quantification of the dispersion of lipid droplets in NIH 3T3 cells, 100 cells were randomly chosen from five different coverslip fields, and the percentages of cells showing dispersed or perinuclear localization were calculated.

### Focus formation assay.

NIH 3T3 cells infected with retroviral particles for *PLIN2* overexpression were diluted 1:5 with noninfected wild-type NIH 3T3 cells. These mixed cultures were seeded with cells at a density of 5 × 10^4^ per well in 6-well tissue culture plates, and the medium was changed every 2 days. After 12 days, cells were fixed in 100% ethanol and were stained with 0.05% crystal violet in 20% ethanol for focus visualization.

### Semisolid-medium growth assay.

Six-well tissue culture plates were coated with a growth medium supplemented with 1.2% agarose to prevent cell adhesion. NIH 3T3 cells infected with retroviral particles for *PLIN2* overexpression were trypsinized, and 5 × 10^3^ cells were resuspended in a medium containing 0.6% agarose. After being plated, colonies were left in culture for 20 days. When applicable, representative colonies were visualized by both phase-contrast and fluorescence microscopy to detect EGFP expression (Axio Observer.Z1 microscope; Carl Zeiss), and the total number of colonies was determined. Colony diameters were determined by measuring image pixels with the aid of a stage micrometer slide.

### Immunohistochemistry.

Human colon adenocarcinoma tissue samples and adjacent normal tissue samples that had been fixed in formalin and embedded in paraffin previously were subjected to immunohistochemistry for PLIN2 (AP125; Research Diagnostics Inc., Flanders, NJ) and Ki-67 (MIB-1; Dako). Tissue-containing blocks were cut into 3-μm slices and were placed in slides treated with 4% (3-aminopropyl)triethoxysilane (APTS) (Thermo Scientific). Then the slides were incubated at 60°C for 16 h, followed by a deparaffinization protocol with five 3-min baths in absolute ethanol and one 10-min bath with water. Antigenic recovery occurred in a citrate solution (pH 6.0) at 98°C for 40 min for PLIN2 and in a pressure cooker with a citrate solution for 3 min for Ki-67. We used the Novolink kit (Novocastra) for signal amplification, following the manufacturer’s instructions. Primary antibodies were incubated for 12 h at 4°C, and after staining, the slices were counterstained with hematoxylin for 30 s, dehydrated in five baths with absolute ethanol, and embedded in five xylol baths. The coverslips were then mounted using Entellan mounting medium (Merck). Immunohistochemistry slides were evaluated independently by three different pathologists.

### Statistical analysis.

Statistical analysis of values from lipid droplet quantification during the progression of NIH 3T3 cells through the cell cycle was carried out using repeated-measures analysis of variance (ANOVA) followed by Bonferroni’s comparison test for multiple comparisons. Two-way ANOVA followed by Bonferroni’s comparison test was used to analyze the dispersion percentages for the subcellular localization of lipid droplets and the values from the quantification of lipid droplets in infected NIH 3T3 cells. Statistical analysis of values from G_1_- or S-arrested cells, and from NIH 3T3 or NIH3T4-H-*rasV12* cells, was carried out using the two-tailed unpaired Student *t* test for single comparisons. A *P* value of <0.05 was considered to be statistically significant.
